# General Clinic Atlanta Medical College

**Published:** 1872-12

**Authors:** Wm. Abram Love

**Affiliations:** Professor of Physiology and Clinical Lecturer on Diseases of the Liver and Kidneys


### GENERAL CLINIC ATLANTA MEDICAL COLLEGE.

SERVICE OF WM. ABRAM LOVE, M. D.,

*Professor of Physiology aud Clinical Lecturer on Diseases of the Liver and Kidneys.*

CASE 1. E. D., æt. 70, laborer, has been suffering from chills and fevers since February last, frequently arrested but to return again at the end of one, two or three weeks; bowels constipated, habitually; tongue furred with more or less of a pectiniform appearance along the edges, red and indicative of malarial poison; pulse soft, quick and not very resisting; his breathing decidedly asthmatic; liver slightly enlarged and somewhat tender on pressure. He complains of more or less uneasiness and pain in the head and along the whole length of the spinal column, and that he is gradually losing the use of his right arm with a tendency in the same direction in the left lower extremity. His soft palate presents the peculiar yellow jaundiced hue indicative of the existence of biliary coloring matter in the blood, not eliminated, but found in all cases of hepatic derangement from malarial poison. He has also excessive tenderness of the hips and knees, and will not tolerate pressure, and his locomotion is materially interfered with from this cause as well as from the general letting down of his system, aside from the paralytic tendency. His skin shows much want of action, especially when we consider the fact that the fever following a chill in intermittents always passes off with diaphoresis. On the paralytic side there is little sensation, pinching or pricking with a sharp instrument producing but slight pain, if any, while on the opposite side there exists more or less hyperæsthesia. With the constipation of the bowels there is general torpor, with tardy functional action; his food is not readily digested and often disagrees with the stomach, producing pyrosis, and as a consequence, his system is broken down from want of nutrition.

In this case, gentlemen, you see supervening on the wane of life the *progressive influence of malarial poison*—the hebdomadal return of chills and fever, hepatic derangement, disturbed digestion, general torpor of visceral functional action, impaired nutrition, spinal paresis amounting to paralysis, more or less complete, though partial, but still sufficient to seriously interfere with both sensation and motion, while the asthmatic disturbance, doubtless, originates in the spinal centres of the pneumogastric and sympathetic nerves, or proceeds from visceral derangement, indigestion, the accumulation of ingesta within the bowels, or faulty nutrition from hepatic derangement and vitiated blood tissue.

These are no unusual complications from malaria, and the indications in the treatment are plain and unmistakable; neutralize the malarial poison, remove the constipation, overcome the visceral torpor, arouse the nerve system, build up the blood tissue and promote nutrition by tonics and a generous diet.

For this purpose we will direct for this man, 1st—

℞.—Quinia sulph., grs. ii.

Ferri sulph., gr. $\frac{1}{4}$.

Ext. belladonna, gr. $\frac{1}{5}$.

Ext. aloe, gr. ss.

In pill *ter dies*, until the constipation has been removed, and then the dose to be given twice or once a day to keep the bowels regulated. 2d—

℞.—Tr. nucis vom., gtt. x. in

Tr. gentiana co., ℥ i., *ter dies*.

The nux vomica to be gradually increased to gtt. xxx, or until its physiological effects are produced on the excito-motor nerve. For the purpose of arousing nervous action and to overcome the tenderness along the spinal column the free application of a mixture of spirits turpentine and sweet oil (equal parts) twice a day, followed by the application of the common sad-iron as warm as it can be borne by the patient. He should have a generous mixed diet, taken in such quantities as can be digested. For the purpose of arousing proper cutaneous action, the salt glove may be resorted to, or the cold shower bath from day to day *when it is found that the system will react readily from the shock*, then stimulating the peripheral nerves and through them the nerve centres.

Mercurials are not admissible in such cases as this, notwithstanding their reputed cholagogue effects. By diminishing the fibrine, they break down the slready vitiated blood-vessels and retard recovery. The muriatic, or nitro-muriatic acids would serve a better purpose, and may be combined with the nux vomica and tincture gentian, should it be deemed advisable in the future treatment of the case.

CASE 2. *Endometritis with Ulceration.*—M. M. F., æt. 26, widow, the mother of two children, the younger over two years old. Since her last *accouchement* she has suffered with more or less pain in the back, a "dragging" weight in the uterine region, tenderness on pressure below the umbilicus, a constant lucorrhœal discharge, purulent, and occasionally of late somewhat colored with blood, her menstruation more profuse as well as more painful than formerly, and continuing from six to eight days, instead of three to four, as was her habit before this trouble commenced. She occasionally passes clots, with pains resembling those of labor, after which she feels better until another gathers, when the pain (expulsive) returns. This condition has continued to increase from month to month until the present time. She occasionally suffers from constipation of the bowels, but in other respects her health has been generally good. She has been under the treatment of physicians who have given her medicines and washes, but has not been benefited by either.

On examinatian, by the touch, the uterus is found more or less engorged, feels heavy and fixed, tender on pressure at the external os, or over the fundus—somewhat lowered, but its axis normal. Through the speculum it presents an engorged supervascular condition—the os discharging purulent matter, lips thickened, with a dark cranberry hue ; the sound enters readily the cavity to the fundus, but on the slightest touch the discharge is colored by exudations from the granular ulcerated surface.

This case presents to you, gentlemen, a well marked case of endometritis, inflammation of the lining membrane of the viscus with ulceration, or the breaking down of the tissue molecule by molecule, and the purulent discharge is but their solution pouring out, now and then mixed with blood exuding from the over-engorged vessels, or granules as they give way from time to time, under hypostalic pressure, or from the solution of the vascular walls. Whether this commenced in the fundus or the cervix, we cannot say, as both are involved, and the inflammation or ulceration may travel the one way or the other until both cavities are involved, as in this case. The indications in treatment are the same in either event, that is, destroy the ulceration and arrest the inflammatory action which has led to it. For this purpose vaginal injections, or "*womb washes*" are of little avail, except so far as they may act upon the vaginal portion of the cervix, and in such a case as this, they would benefit only to a limited extent. Intra-uterine medication, direct applications to the inflamed and ulcerated surfaces promises the only relief. This may be done by the introduction of solids or fluids into the uterine cavity by proper instruments, and the results, with proper manipulation and due precaution, are usually satisfactory. These manipulations require a nice sense of touch and a watchful care for an organ that, while it seems, and is, in one sense, insensible, is yet the very centre of all sensations, so to speak, and hasty action and the incautious use of medicines endometrically, may give you occasion and *leisure* to repent. This is particularly the case with regard to intra-uterine injections. Their introduction will sometimes be followed, where you least expect it, by a train of symptoms, nervous but severe and alarming in character, that will deter you thenceforth from their use, and learn you *never to use an intra-uterine injection of any kind unless the internal os uteri has been dilated, and not then unless its temperature is near that of the blood.* They induce spasmodic contraction of the transverse and other fibres and in this those around the internal os close first, and the fluids may be forced through the fallopian tubes in the peritoneal cavity and bring rapidly peritonitis, aside from the alarming nervous symptoms more immediately induced.

Medicines may be as effectually and advantageously applied—fluids or solids—by the probe, probang, porte caustique, or medicated tent, etc., without the risk of "uterine colic," at least to so great an extent, and for this reason we should prefer them, particularly when we have not the time nor the appliances for dilating the organ as a precautionary and preparatory measure.

In this case the internal os admits of the passage of a Simpson's sound readily; and with this an application will be made of the "*Reduced Nitrate of Silver*" in the form of powder (Arget: Nit. cum Pot.: Nit.-partes equalis; fused and powdered.) The sound, wrapped for two and a half inches with cotton lint, is moistened with lard, oil or water, dipped into the powder. A quantity adheres, and is inserted into the uterine cavity, and allowed to remain for a moment or two.) The introduction of the medicated cotton-bound sound induces always contraction, whatever be the agent used; hence it should never be forcibly removed; better to wait awhile, until the spasmodic contraction ceases, when relaxation follows, than to remove rapidly, and injure the tissues in the act.

This application should be repeated about once every six or seven days, or substituted for the iodine solution—glycerine and carbolic acid—or the muriated tincture of iron, as occasion may require or the conditions indicate. To aid in the further reduction of the uterine engorgement, a tampon of cotton lint (to which a thread for its removal is attached), saturated with glycerine, is inserted against the os uteri and in the cul-de-sac, and may be renewed from day to day. The affinity of the glycerine for water drains, by exosmotic action, a large amount of water from the congested organ, reduces the weight and engorgment, takes off the pressure on the vessels, and materially aids in reducing the inflammation, while it at the same time prevents further putrefaction in the purulent discharges. To further aid in allaying irritation, much benefit may be derived from the internal use of the bromide of potassium, with or without opium or its preparations; better in these cases to combine it with valerian and ammonia; and we find an excellent combination in the officinal ammoniated tincture, when added to a solution of the bromide. These aids to treatment should never be overlooked. Any interference with the internal uterus induces excessive nervousness, and this should be controlled for the comfort and well-being of your patient. With these views we shall direct—

℞.—Tr. ammoniæ valeri, ʒ i.

Cum pot. brom. grs. x.

In aq. f., ℥ ii.

Ter die, or pro re nata.

To allay nervous disturbance and quiet any excitement about the uterus or ovaries.

Case 3. *Influenza and pleurodynia.*—J. D., colored, æt. 50, male, laborer, well-developed, muscular; health has been good generally; five days since, on ceasing work, after much fatigue, and while in a free perspiration, on striking the cold took a chill, with a severe cold, pain in the head and back, sore throat—first in the pharynx, then extending down the bronchial tubes, from day to day, with cough—when yesterday he was attacked with severe pain in the left side; an effort at deep inhalation increases its severity very much, and the least pressure on the intercostal spaces is unbearable. There is very little bronchial secretion. He has fever; skin dry, tongue furred with a brown coat, bowels constipated, soft palate, yellow, tenderness over the liver, respiration humid and painful, some pain in his shoulder joint on moving the arm, but suffers most from pain in side when coughing.

This, gentlemen, is but a specimen of the cases frequently seen in the city, amounting almost to an epidemic, with the recent sudden changes in the weather; a species of influenza, from some subtle cause not at all incompatible with malaria, because those who are suffering from the effects of malarial poison seem most speedily as well as most severely attacked with the disease.

It would seem to have its origin in some peculiar electrical condition of the atmosphere, and to produce, with its progressive catarrhal irritation or inflammation of the mucous membrane of the air passages, also, more or less disturbance of the serous cavities, as you see in the case before you. In some cases it appears to attack the serous membrane first, then fall back on the mucous linings. Generally it commences in the upper portion of the air passages, and progresses through to the minute ramification of the bronchial tubes, then attacking the pleura, costal or pulmonary, or both, or culminating in pleurodynia, as you see here. At other times it passes on to the air vesicles, causing pneumonia. Or, again, it takes a longer leap, and attacks the joints, one or many, and resembles neuralgic rheumatism. Where it attacks these it would seem to partake more of the character of peripheral nerve paresis than tissue irritation in the outset, but continuing, both irritation and inflammation may supervene at these several points, while there is no modification of the pharyngeal, laryngeal, or bronchial symptoms. In some cases the central and spinal meninges become affected, and in a more malignant form would develop cerebro-spinal meningitis. In the mild form in which it has presented itself so far, it has proved tractable, and yields kindly to judicious treatment in a few days, but with more or less tendency to return, from slight exposures. Anodynes, particularly opiates, with expectorants, together with warm applications and counter-irritants over the seat of pain, generally produce the desired effect. In this case, presenting some complications not usually found, we will direct—

℞.—Pv. ipecac et opii, grs. x.

Hyd. chl. mit, grs. iij.

Every four hours, until three doses are taken; to be followed by a cathartic of ol. ricini ℥ i., ol. terebinth gtt. xx. to-morrow morning; also a sinapism, to be made up with syrup, and worn over the left lung. As an expectorant we will give him—

℞.—Syr. senega, ʒ i.

Syr. ipecac, ʒ i.

Tr. opii camph., ʒ i.

Every four hours with Dover's powder, grs. x, at bed-time and direct him to return to-morrow.

These cases yield readily to the opium treatment, when given in sufficient doses to impress the nerve system, and while the Caucasian, as a general rule, will require a much larger dose to produce a given effect, those of African descent go readily under its influence with the minimum doses, otherwise we should have directed it in larger portions.

*Second day.*—Better; the pleurodynia nearly subsided, bronchial symptoms improving, cathartic acted well, producing copious bilious discharges. Continued expectorant and anodyne at bedtime.

*Fourth day.*—Still improving. Continued treatment.

*Sixth day.*—Reported entirely relieved, and discharged.

CASE 4. *Chills and Fever–Chronic.*—R. H., male, æt. thirty-four, colored, laborer; has been a stout, healthy man, but for the past six years has been subject to chills and fever every fall, following him into the winter months. He had a chill every other day until recently, when they commenced returning every third day. There is slight enlargement of the liver and spleen, torpor of the bowels, and though the eyes are clear of tinge, the soft palate presents the golden hue found in such cases from the coloring matter of bile. There are no other prominent symptoms presented in this case, more than you find in an ordinary case of chills and fever. His appetite is variable, sometimes morbidly increased, at others he loathes food, and his digestion, as a matter of course, keeps apace.

You will find these cases, gentlemen, in your future experience, obstinate and intractable, and you will often have to attack by seige and by storm, and when you think you have effectually routed him, at the end of some multiple of seven days, some hebdomadal period, find that he is still your enemy. Nothing but persistence, for weeks and weeks, in the use of tonics and anti-periodics will eradicate the disease, and should you stop short of this, you will have occasion to regret so doing; better not take upon yourself the responsibility of the treatment of such a case, than to stop your remedies at too early a period. If your patients will not promise you to persist in treatment until the disease is eradicated, you had best take care of your own reputation and let them alone, otherwise you are wasting your time and your medicine, as well as the health, the constitution and the substance of your patients, and it will rebound on you ultimately.

For this patient we shall direct—

℞.—Pil. mass hydrarg., grs. iij.
Quinia sulp., grs. iij.
Pv. capsici, gr. ss.

In pills, every two hours until six pills are taken, commencing twelve or fourteen hours before each chill, to be followed by a laxative, if necessary. The same to be repeated until the soft palate has cleared up, unless the unconstitutional effects of the mercury should deter from its further use; and as part of the treatment—

℞.—Ext. nucis vom. grs. ¼.

Ext. belladon. gr. ⅙.

Acid arsenious, gr. 1-24.

Quinia sulph., grs. i.

Ferri. proto-carb., grs. ij.

In pil, ter die, unless the arsenious acid should puff the face, when it should be omitted. The anti-intermittent tonic should be continued for several weeks, gradually dropping the modicum down to two or one pill per diem, or at longer intervals, that the system may be brought from under the influence of the medicine. Counter-irritation to the spine, friction over the surface, cold sponging, the shower-bath, may materially aid in arousing the depressed nerve forces, and a generous, digestible and proper diet to build up the tissues, should in no case be neglected.

